# miRNA profiling in metastatic renal cell carcinoma reveals a tumour-suppressor effect for miR-215

**DOI:** 10.1038/bjc.2011.401

**Published:** 2011-10-27

**Authors:** N M A White, H W Z Khella, J Grigull, S Adzovic, Y M Youssef, R J Honey, R Stewart, K T Pace, G A Bjarnason, M A S Jewett, A J Evans, M Gabril, G M Yousef

**Affiliations:** 1 Department of Laboratory Medicine and the Keenan Research Centre in the Li Ka Shing Knowledge Institute of St. Michael's Hospital, Toronto, ON, Canada, M5B 1W8; 2 Department of Laboratory Medicine and Pathobiology, University of Toronto, Toronto, ON, Canada, M5S 1A8; 3 Institute of Medical Sciences, University of Toronto, Toronto, ON, Canada, M5S 1A8; 4 Department of Mathematics and Statistics, York University, Toronto, ON, Canada, M3J 1P3; 5 Department of Surgery, St. Michael's Hospital, Toronto, ON, Canada, M5B 1W8; 6 Division of Medical Oncology and Hematology, Sunnybrook Health Sciences, Toronto, ON, Canada, M4N 3M5; 7 Department of Surgery, Princess Margaret Hospital, Toronto, ON, Canada, M5G 2M9; 8 Department of Pathology, London Health Sciences Center and University of Western Ontario, London, ON, Canada, N6A 5W9

**Keywords:** clear cell renal cell carcinoma, kidney cancer, metastasis, microRNA, miR-215, tumour markers

## Abstract

**Background:**

Renal cell carcinoma (RCC) is the most common neoplasm of the adult kidney. Metastatic RCC is difficult to treat. The 5-year survival rate for metastatic RCC is ⩽10%. Recently, microRNAs (miRNAs) have been shown to have a role in cancer metastasis and potential as prognostic biomarkers in cancer.

**Method:**

We performed a miRNA microarray to identify a miRNA signature characteristic of metastatic compared with primary RCCs. We validated our results by quantitative real-time PCR. We performed experimental and bioinformatic analyses to explore the involvement of miR-215 in RCC progression and metastasis.

**Results:**

We identified 65 miRNAs that were significantly altered in metastatic compared with primary RCCs. We validated our results by examining the expression of miR-10b, miR-126, miR-196a, miR-204 and miR-215, in two independent cohorts of patients. We showed that overexpression of miR-215 decreased cellular migration and invasion in an RCC cell line model. In addition, through gene expression profiling, we identified direct and indirect targets of miR-215 that can contribute to tumour metastasis.

**Conclusion:**

Our analysis showed that miRNAs are altered in metastatic RCCs and can contribute to kidney cancer metastasis through different biological processes. Dysregulated miRNAs represent potential prognostic biomarkers and may have therapeutic applications in kidney cancer.

Renal cell carcinoma (RCC) is the most common neoplasm of the adult kidney. The overall incidence and mortality of RCC have increased over the past 20 years (Hollingsworth *et al*, 2006). Approximately 75–80% RCC patients are diagnosed with the clear cell RCC (ccRCC) subtype (Capitanio *et al*, 2009). Although surgery is curative for localised disease, a significant proportion of patients relapse or metastasize. Metastatic RCC is difficult to treat. There have been many advances in RCC-targeted therapy in recent years, but unfortunately, despite these advances, the majority of patients ultimately still progress and succumb to the disease. The 5-year survival rate for metastatic RCC is dismal at ⩽10% (Motzer *et al*, 1996; Tsui *et al*, 2000). Predicting recurrences early can impact patient outcome as the likelihood of a favourable response to treatment is greater with limited metastatic burden (Antonelli *et al*, 2007).

The process of metastasis is not well understood. Although quite a complex process, it can be summarised in a series of steps starting with local invasion, followed by intravasation, survival in the circulation, extravasation, initiation and maintenance of micrometastasis at a distant site and finally, vascularisation of the new tumour (Gupta and Massague, 2006). Recently, microRNAs (miRNAs), small non-protein-coding RNAs, have been reported to be involved in cancer progression and metastasis (Le *et al*, 2010; White *et al*, 2011b). MicroRNAs are approximately 19–25 nucleotides in length and have been found to negatively regulate gene expression at the post-transcriptional level by binding to target mRNAs leading to mRNA degradation or translational repression (He and Hannon, 2004).

Accumulating evidence shows that miRNAs have a role in the pathogenesis of RCC (Schaefer *et al*, 2010b). Recently, we along with others have reported dysregulation of miRNA expression in kidney cancer (Nakada *et al*, 2008; Jung *et al*, 2009; Juan *et al*, 2010; Youssef *et al*, 2011; White *et al*, 2011a). MicroRNAs have been shown to have an oncogenic effect on RCC (Chow *et al*, 2010a). As recently highlighted, miRNAs can contribute to RCC pathogenesis at different levels (White and Yousef, 2010). Several miRNAs are downstream effectors of the hypoxia-induced factor (HIF)-induced hypoxia response. In addition, certain miRNAs are regulated by von Hippel-Lindau in either a HIF-dependent or HIF-independent manner in RCC (Neal *et al*, 2010). Apart from the hypoxia pathway, recent experiments have shown that miRNAs can directly affect tumour proliferation and apoptosis through mechanisms that are yet to be identified (White *et al*, 2010a; Chow *et al*, 2010b).

miRNAs have also been shown to be involved in tumour progression and metastasis in colon (Huang *et al*, 2009), kidney (Heinzelmann *et al*, 2011) and other cancers. In a recent study, miRNAs were shown to stimulate breast cancer cell migration and invasion both *in vitro* and *in vivo* (Huang *et al*, 2008). In colon cancer, high miRNA expression was shown to promote cancer cell detachment, migration and invasion (Schimanski *et al*, 2009). Understanding the role miRNAs in metastasis will help elucidate this complex process and may lead to novel miRNA therapeutic targets for prevention or treatment of RCC metastasis.

MicroRNAs have also been shown to have clinical utility as cancer biomarkers (Arsanious *et al*, 2009; Metias *et al*, 2009; Schaefer *et al*, 2010a; Youssef *et al*, 2011). MicroRNAs were shown to be able to differentiate between primary lung cancer and lung metastasis (Barshack *et al*, 2010) and to be independent markers for poor prognosis in patients with lung and head and neck cancers (Childs *et al*, 2009; Gallardo *et al*, 2009).

In this study, we performed a miRNA microarray to analyse the expression profile of metastatic compared with primary ccRCC tumours. We validated our results by quantitative real-time PCR in two independent cohorts of patients. We also examined the role of one of the top dysregulated miRNAs, miR-215, in the pathogenesis of RCC metastasis. Our results showed that miR-215 is involved in cancer cellular migration and proliferation. We also identified a number of direct and indirect targets that are affected by overexpression of miR-215.

## Patients and methods

### Patient specimens

Patient specimens, including 28 fresh frozen and a total of 80 (both primary and metastatic) formalin-fixed paraffin-embedded (FFPE) tissues, were obtained from St Michael's Hospital, University Health Network, London Health Sciences Center and the Ontario Tumor Bank. Fresh specimens were collected immediately after resection, snap frozen in liquid nitrogen and stored at −80 °C until total RNA extraction. Samples were taken from areas with no haemorrhage or necrosis, and multiple sections were submitted from the same tumour to compensate for tumour heterogeneity. All diagnoses were confirmed by two pathologists. All procedures were carried out in accordance with St Michael's Research Ethics Board.

### Total RNA extraction and miRNA microarray

In all, 2 mg each of fresh-frozen primary and metastatic ccRCC tissues were used for nucleic acid isolation. Total RNA was extracted using the miRNeasy kit (Qiagen, Mississauga, ON, Canada) according to the manufacturer's protocol. Total RNA was extracted from cores of FFPE samples using the miRNeasy FFPE kit (Qiagen) according to the manufacturer's protocol. Total RNA concentrations were determined spectrophotometrically, and the quality of extracted RNA was assessed by electropherogram and gel analysis. Samples suitable for analysis were stored at −80 °C.

Microarray analysis was performed on 4 *μ*g of total RNA obtained from 18 fresh-frozen primary ccRCC and 10 unmatched metastatic RCC tumours and was carried out using the *μ*Paraflo microfluidic technology, as per the manufacturer's protocol (LC Sciences, Houston, TX, USA). Hybridisation was performed overnight on a microfluidic chip. The array assessed all miRNA transcripts available in the version of the Sanger miRBase database (version 13.0) available at the time of the array. Post-hybridisation detection used fluorescence labelling with tag-specific Cy3 and Cy5 dyes. Hybridisation images were collected using a GenePix 4000B laser scanner (Molecular Device, Sunnyvale, CA, USA) and digitised using Array-Pro image analysis software (Media Cybernetics, Bethesda, MD, USA).

### Quantitative real-time RT–PCR

Quantitative real-time PCR (qRT–PCR) was used to measure miRNA expression using TaqMan MicroRNA assays (Applied Biosystems, Foster City, CA, USA) as described in our recent publications (Youssef *et al*, 2011). MicroRNA-specific reverse transcription was performed with 5 ng total RNA using the TaqMan MicroRNA reverse transcription kit (Applied Biosystems) as recommended by the manufacturer for miR-10b, miR-126, miR-196a, miR-204 and miR-215. Quantitative real-time PCR was performed using the TaqMan microRNA assay kit on the Step-One Plus Real-Time PCR System (Applied Biosystems). Thermal cycling conditions were according to the manufacturer's fast protocol and all reactions were performed in duplicate. Relative expression was determined using the ΔΔC_T_ method and expression values were normalised to small nucleolar RNA, C/D box 44 (*SNORD44*, also known as *RNU44*), which has been shown to be a suitable reference gene (Chow *et al*, 2010a).

### Cell culture and miRNA transfection

786-O and CAKI-1 RCC cells were obtained from American Type Culture Collection (ATCC, Manassas, VA, USA). 786-O cells were maintained in Dulbecco's modified Eagle's medium (DMEM) supplemented with 10% fetal calf serum (FCS). CAKI-1 cells were maintained in McCoy's 5A medium supplemented with 10% FCS. All cells were maintained in 5% CO_2_ at 37 °C.

Pre-miR precursors and anti-miR miR inhibitors (Applied Biosystems) were purchased for miR-215. Cells were transfected using siPORT *NeoFX* transfection agent (Ambion, Austin, TX, USA) as recommended by the manufacturer and described in previous publications (Chow *et al*, 2010a; White *et al*, 2010a, 2010b). In brief, siPORT *NeoFX* transfection agent was diluted in Opti-MEM reduced serum media (Invitrogen, Carlsbad, CA, USA). Complexes were allowed to form for 10 min at room temperature. Precursor miRNA and miRNA inhibitors were diluted in Opti-MEM reduced serum media, combined with siPORT NeoFX, and incubated for 10 min at room temperature. Transfection complexes were added to the cell culture plate and overlayed with cell suspensions. Cells were then incubated at 37 °C and 5% CO_2_. The final concentration of the miRNA precursor or inhibitor was 30 nM.

### Wound-healing assay

786-O cells were plated at 8.0. × 10^4^ cells per well in a 12-well plate and transfected with miR-215, anti-miR-215 or co-transfected with miR-215 and its inhibitor as described above. Twenty-four hours later, the cell monolayer was wounded with a 200 *μ*l pipette tip. Photomicrographs were taken at the time of wounding (0 h) and 11 h later. The rate of migration is displayed as the percentage of cell covered area (100 – percentage cell-free area) where percentage cell-free area is defined as ((cell-free area_11 h_/cell-free area_0 h_) × 100). The area was measured using the Image J Software (National Institutes of Health, Bethesda, MD, USA, http://rsbweb.nih.gov/ij/). Cells were transfected in three separate transfections and each was analysed in triplicate. Three images were taken per ‘wound’. A gridded coverslip was attached to the bottom of each well to ensure photomicrographs were being taken at the exact place each time.

### Invasion assay

The effect of miR-215 on cellular invasion was examined using BD BioCoat Matrigel Invasion Chamber (BD Biosciences, Bedford, MA, USA). 786-O cells were transfected with miR-215, anti-miR-215 or co-transfected with miR-215 and its inhibitor. Twenty-four hours after transfection, cells were trypsinised and resuspended in DMEM supplemented with 0.5% FCS. A total of 5.0 × 10^4^ cells were plated on the upper chamber. A normal growth medium, DMEM supplemented with 10% FCS, was added to the bottom chamber as a chemoattractant. Cells were allowed to invade for 22 h. After incubation, non-invading cells were removed from the upper surface and cells on the lower surface were stained with Diff-Quick. Invading cells were counted by taking photomicrographs at × 40 magnification in 3 fields. The experiment was performed in triplicate. Cell invasion is displayed as % invasion (mean number of cells that invaded through Matrigel insert/mean number of cells that migrated through the control insert membrane).

### Cell proliferation assay

Cellular proliferation was measured by both the 3-(4,5-dimethylthiazol-2-yl)-2,5-diphenyltetrazolium bromide (MTT; Roche, Mississauga, ON, Canada) assay and cell counting. 786-O cells were plated at 6.0 × 10^3^ cells per well in a 96-well plate and transfected with miR-215, anti-miR-215 or co-transfected with miR-215 and its inhibitor. Cells were incubated for 1–4 days after which 10 *μ*l of 5 mg ml^−1^ solution of MTT in phosphate-buffered saline was added and incubated for 4 h at 37 °C. The precipitate was then solubilised in 100 *μ*l solubilisation solution (10% SDS in 0.01 M HCl) and incubated at 37 °C overnight. The absorbance of each well was measured at a wavelength of 550 nm. Each test was repeated in triplicate. Cell proliferation was also assayed by cell counting. 786-O cells were plated at 8.0 × 10^4^ cells per well and were untransfected or transfected with miR-215, anti-miR-215 or co-transfected with miR-215 and its inhibitor. Cells were incubated for 1–4 days after which they were trypsinised and counted. Cells were counted in triplicate and repeated thrice.

### Western blot

Cells were lysed 48 h after transfection using NETN lysis buffer (0.5% Nonidet-P40, 100 nM NaCl, 1 mM EDTA and 20 mM Tris-Cl (pH 8.0) with protease inhibitor cocktail tablets (Roche)) and cleared by centrifugation at 21 000 **g** for 10 min at 4 °C. Protein concentrations were determined using the BCA protein assay reagent (Pierce Biotechnology, Rockford, IL, USA) using bovine serum albumin (BSA) as a standard.

Total cellular protein was separated in 12% SDS–PAGE and transferred to a nitrocellulose membrane. The membrane was blocked with 3% BSA in TBST (150 mM NaCl, 10 mM Tris-HCl pH 8.0 and 0.1% (v/v) Tween 20) and incubated with anti-SIP1/ZEB2 primary antibody (1 *μ*g ml^−1^, Abcam, Cambridge, MA, USA) diluted in blocking solution overnight at 4 °C with shaking. The membrane was then washed with TBST, incubated with anti-rabbit horseradish peroxidase-conjugated secondary antibody (Santa Cruz Biotechnology, Santa Cruz, CA, USA) diluted in blocking solution for 1 h. Immune complexes were visualised using enhanced chemiluminescence and Hyperfilm (GE Healthcare, Piscataway, NJ, USA). Membranes were stripped and re-probed for *β*-actin (1 : 1000, Cell Signalling Technology Inc., Danvers, MA, USA) as a loading control.

### RT^2^ tumour metastasis profiler assay

CAKI-1 cells were transfected with miR-215 as described above and total RNA was collected after 48 h. Genomic DNA contamination was eliminated using the RNase-Free DNase Set (Qiagen). For PCR array experiments, the RT^2^ Profiler Tumor Metastasis PCR Array (SA Biosciences, Frederick, MD, USA) was used to simultaneously examine the levels of 89 genes associated with metastasis. In brief, cDNA was synthesised from 1 *μ*g total RNA using the RT^2^ First-Strand Kit (SA Biosciences) according to the manufacturer's protocol. Reaction mixtures were incubated at 42 °C for 15 min, 95 °C for 5 min and held on ice until the PCR reaction. Changes in expression were measured by obtaining the threshold cycle and normalising to the average of five housekeeping genes. Fold change was calculated by 2^(−ΔΔCt)^ where ΔΔC_t_=(CAKI-1 cells transfected with miR-215 (C_t_ target−C_t_ control))−(untransfected CAKI-1 cells (C_t_ target−C_t_ control)).

### Statistical and clustering analysis

LOWESS regression was applied to normalise raw quantitated intensities from the miRNA microarray analysis. Expression data from 875 miRNAs in 18 primary cancer and 10 (unpaired) metastatic cancer tissues were collated and filtered using Class Comparison in BRB ARRAYTools (version 3.8, developed by Dr Richard Simon and BRB-ArrayTools Development Team) and intensities were thresholded to 500. Using a false-discovery rate of 20% at a confidence level of 90%, 65 miRNAs were identified as differentially expressed between metastatic and primary tissues. Significantly dysregulated miRNAs were clustered using the EisenLab Cluster and TreeView software (accessed through BRB ARRAYTools).

All other statistical analyses were performed using GraphPad Prism 5 Software (GraphPad, La Jolla, CA, USA). One-way ANOVA and *post hoc* multiple comparisons (Tukey's) were used to compare differences in mRNA expression, wound-healing and invasion assays. A *P*-value <0.05 was considered significant.

### Bioinformatics analysis

miRNA target prediction analyses were performed using two programs; Target Combo ‘Predicted Targets: Union’ (Sethupathy *et al*, 2006) and miRecords (Xiao *et al*, 2009). A prediction was only included if it was detected by at least four programs.

## Results

### miRNA microarray

**Figure 1:**
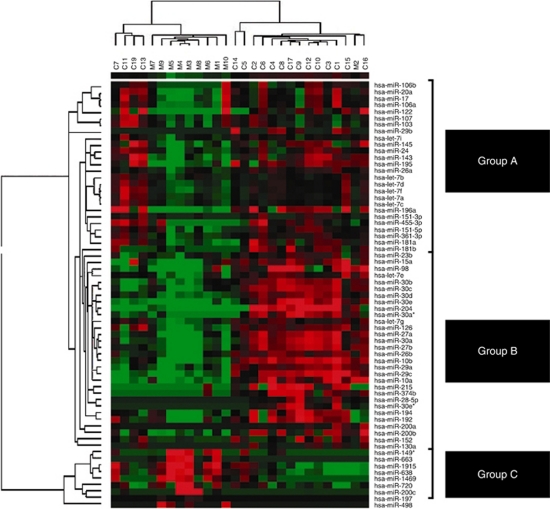
Heat map showing the top statistically significant (*P*<0.05) altered miRNAs in metastatic (m) compared with primary (c) ccRCC. A non-supervised analysis was applied to 10 metastatic RCC and 18 primary ccRCC profiles for 65 statistically significant miRNAs. Intensity profiles are displayed as a tree on the top and miRNA reporters are displayed on the right of the heat map with the tree on the left. Upregulation in metastasis is depicted as red squares and downregulation as green squares. The colour reproduction of this figure is available at the *British Journal of Cancer* online.

Microarray analysis was performed on a total of 18 primary ccRCCs and 10 unmatched metastatic fresh-frozen tumours. In total, expression values for 875 miRNAs were reported. We identified 65 miRNAs that were significantly dysregulated in metastatic when compared with primary RCCs (Table 1). In all, 9 (14%) had increased expression, whereas 56 (86%) had decreased expression. miR-10b, miR-196a and miR-27b were the most downregulated, whereas miR-638, miR-1915 and miR-149^*^ were the most upregulated in metastasis when compared with primary RCCs. A non-supervised 2D-cluster analysis was applied for all tumours and the 65 significantly dysregulated miRNAs (Figure 1).

We identified a miRNA signature that can reliably distinguish between primary and metastatic tumours. Interestingly, a subgroup of the primary tumours (C7, C11, C19 and C13) clustered under the metastatic arm with a group of miRNAs that follow the same pattern of expression (Figure 1, Group B), suggesting that they have an inherited aggressive signature. The dysregulated miRNAs can be clustered into three groups. Group A shows miRNA downregulation in metastasis when compared with primary (with the exception of one metastatic tumour). Group B shows a much more distinct upregulation of miRNAs in primary tumours and downregulation in both metastatic and the subgroup of primary tumours mentioned above. Group C shows miRNA downregulation in primary and upregulation in metastatic tumours.

### Quantitative real-time PCR validation

**Figure 2:**
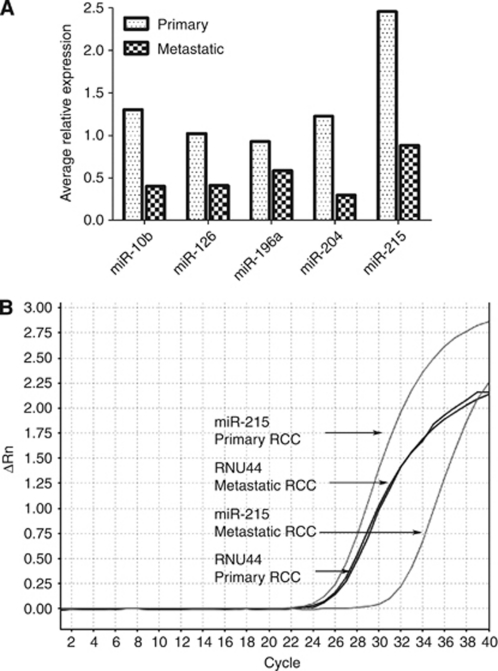
Quantitative real-time PCR validation of miRNA microarray analysis. (**A**) Bar graph showing average expressions of miR-10b, miR-126, miR-196a, miR-204 and miR-215 were decreased in metastatic when compared with primary RCC. (**B**) Representative real-time PCR amplification plot showing miR-215 downregulation in metastatic compared with primary RCCs.

On the basis of miRNA microarray results, we experimentally verified expression levels of five miRNAs, miR-10b, miR-126, miR-196a, miR-204 and miR-215, in two independent cohorts of tissues with the ‘gold-standard’ qRT–PCR using miRNA-specific TaqMan probes. First, we verified our results on 18 primary and 10 metastatic unmatched fresh-frozen RCC tissues. As shown in Figure 2A, all five miRNAs showed decreased expression in metastatic when compared with primary RCCs. These results are comparable to expression levels of the microarray analysis. We also validated our results on a separate cohort of 40 primary RCCs and 40 unmatched RCC metastatic FFPE tissues. All miRNAs showed decreased expression in the metastatic tumours, thus further validating both the microarray analysis and the fresh-tissue PCR analysis. A representative amplification plot of miR-215 expression is shown in Figure 2B.

### Bioinformatics analysis

We further explored the role of these dysregulated miRNAs in RCC tumour progression and metastasis through bioinformatics analysis. Interestingly, a literature search showed that many of the miRNAs that we found dysregulated in metastatic RCCs have also been reported to be altered in tumour progression and metastasis of other cancers (Table 2). This implies the presence of common mechanisms/pathways that are used by multiple malignancies to achieve a metastatic phenotype. We also performed target prediction analysis for these miRNAs. As shown in Supplementary Table 1, a number of critical molecules that are documented to have a role in metastasis are among the predicted targets of miRNAs, namely MMP3, TIMP3 and SIP1/ZEB2.

### miR-215 overexpression slows cellular migration

To investigate the role of miRNAs in tumour metastasis, we chose to further characterise the effect of miR-215 in a RCC cell line model. Our analysis showed that miR-215 was among the most significantly downregulated miRNAs in metastatic when compared with primary RCC tumours (Table 1). In addition, miR-215 was dysregulated in the vast majority of metastatic tumours analysed (downregulation was reported in 90% samples in the microarray, 100% fresh frozen samples and 87% FFPE samples analysed by qRT–PCR, data not shown). miR-215 was also chosen for further characterisation based on its reported dysregulation in other cancers, its known biological effect in other cancers and the fact that one of its targets, SIP1/ZEB2, has been shown to be involved in epithelial-to-mesenchymal transition.

**Figure 3:**
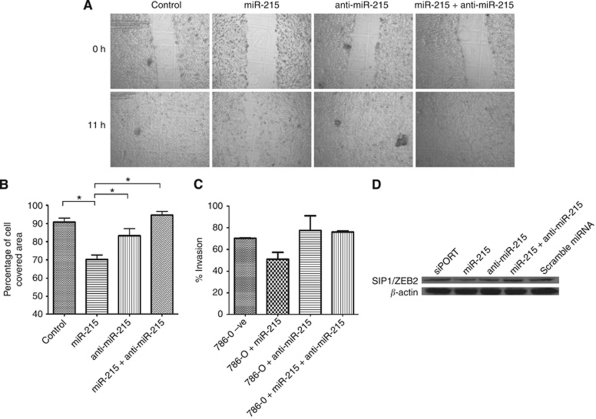
miR-215 has a negative effect on cellular migration and invasion. (**A**) Representative photomicrographs of untransfected 786-O kidney cancer cells (Control) and 786-O cells transfected with miR-215, anti-miR-215 or a combination of both at the time of wounding (0 h) and 11 h later. All photomicrographs were taken at × 40 magnification. (**B**) Cell migration was measured by comparing the cell covered area at the time of wounding (0 h) and 11 hours later (11 h). Cells transfected with miR-215 (70% cell covered area) showed a significantly slower migration rate than did control cells (90%), cells transfected with anti-miR-215 (86%) and cells transfected with a combination of miR-215 and anti-miR-215. (**C**) 786-O cells were transfected with miR-215, anti-miR-215 or a combination of miR-215 and its inhibitor. Cells transfected with miR-215 showed decreased cellular invasion after 22 h compared with those transfected with anti-miR-215 or a combination of miR-215 and its inhibitor. (**D**) 786-O cells were transfected with transfection reagent alone, miR-215, anti-miR-215, a combination of miR-215 and its inhibitor, or a random sequence miRNA, and the expression of SIP1/ZEB2 was examined by western blot analysis. There was decreased protein expression in cells that were transfected with miR-215. All other conditions showed no change in protein expression. *β*-Actin was used as a loading control. ^*^P<0.05.

We analysed the effect of miR-215 on cellular migration by a wound-healing assay. Representative microphotographs of the assay are shown in Figure 3A. When the 786-O kidney cancer cell line, which has no endogenous miR-215 expression, was transfected with miR-215, cells migrated significantly slower than did control cells (70 *vs* 90% cell covered area, respectively, *P*<0.05), cells transfected with the miR-215 inhibitor (70 *vs* 86% cell covered area, respectively, *P*<0.05) and cells transfected with a combination of the miR-215 and its inhibitor (70 *vs* 94% cell covered area, respectively, *P*<0.05; Figure 3B), indicating that miR-215 has a negative effect on cellular migration.

### miR-215 decreases cellular invasion

The effect of miR-215 on cell invasion was measured through a cellular invasion assay as described in the ‘Patients and methods’ section. 786-O cells were transfected with miR-215, its inhibitor, or a combination of both and allowed to invade through Matrigel for 22 h. The number of cells that invaded was compared with control-untransfected cells. Cells that were transfected with miR-215 showed a decrease in cellular invasion (Figure 3C). Cells that were transfected with either the anti-miR-215 or a combination of miR-215 and its inhibitor showed invasion levels that were comparable to control cells.

### miR-215 affects cell proliferation

To see the effect of miR-215 on cellular proliferation, we performed an MTT assay. 786-O cells were transfected with miR-215 or anti-miR-215 and cell proliferation was measured over 4 days. Increased miRNA expression was sustained for at least 5 days after transfection (data not shown). Cells transfected with miR-215 showed a slight decrease in cell proliferation when compared with control cells (Supplementary Figure 1A). To confirm these results, we assayed cell proliferation by cell counting. The RCC cells that were transfected with miR-215 showed decreased cell growth compared with untransfected cells (Supplementary Figure 1B). To see the specific effects of increased miR-215 expression on cell cycle, we analysed cell distribution by propidium iodide staining. When cells were transfected with miR-215, there was a slight increase in the number of transfected cells in the G1 phase (49% *vs* 51%) and a decrease in cells in the G2/M phase (22% *vs* 21%) phase (data not shown).

### miR-215 can directly target SIP1/ZEB2

To explore the mechanism by which miR-215 negatively affects cell migration and invasion, we examined the effect of miR-215 transfection on SIP1/ZEB2 (smad-interacting protein 1/zinc-finger E-box-binding homeobox 2) protein expression, which is a direct predicted target of miR-215 (Supplementary Table 1). SIP1/ZEB2 is a member of the *δ*EF-1 family of zinc nuclear factors and represses E-cadherin. It has been shown to be involved in epithelial-to-mesenchymal transition, and recent evidence showed that its expression can be regulated by miRNAs (Park *et al*, 2008). 786-O cells were transfected with miR-215, anti-miR-215 or a combination of both and SIP1/ZEB2 protein expression was examined. There was a decrease in SIP1/ZEB2 protein expression when cells were transfected with miR-215 (Figure 3D), confirming that SIP1/ZEB2 is a direct target of miR-215. There was no change in SIP1/ZEB2 expression when cells were transfected with the miR-215 inhibitor, a combination of the miR-215 and its inhibitor, or scramble miRNA.

### The effect of miR-215 on downstream targets involved in metastasis

We performed target prediction analysis to identify potential targets of miR-215 that can affect metastasis as shown in Supplementary Table 1. To identify potential metastasis-related pathways that can be regulated by miR-215, the effect of miR-215 transfection on the expression levels of a panel of 89 genes that are documented to be altered in metastasis was measured in a kidney cancer cell line model using a ‘Tumor Metastasis PCR Array.’ We identified 27 genes that had a fold regulation ±1.2 upon miR-215 transfection (Table 3). Interestingly, two of these genes, EPH receptor B2 (*EBHB2*) and nuclear receptor subfamily 4, group A, member 4 (*NR4A3*), which both show gene downregulation (−1.043 and −1.257, respectively), are direct predicted targets of miR-215 and are involved in cellular proliferation. Other genes involved in cell proliferation that had decreased expression were *MYC*, *IGF1* and *SMAD2*. We also found changes in gene expression in cell adhesion genes, including decreased *cadherin 6* and *11* and *MMP3* and *HPSE*.

## Discussion

In this study, we identified a miRNA signature for metastatic compared with primary RCCs. The majority (86%) of the miRNAs identified were downregulated. This trend of miRNA downregulation in metastasis has also been reported for other cancers (Zhang *et al*, 2010). An interesting observation is that a number of miRNAs that were reported to be downregulated in primary RCCs (White *et al*, 2011a) were reported to be further downregulated in metastatic RCCs, suggesting that these miRNAs may contribute to both the initiation and progression of RCC and that their progressive downregulation may have a further role in the development of metastatic RCC. For example, miR-204 was reported to be downregulated in primary RCCs and was found to be further downregulated in the current analysis.

Although we identified many unique miRNAs that are significantly dysregulated in metastatic RCCs, a number of miRNAs identified were also reported in other metastatic cancers. For example, miR-122 and miR-126 (Budhu *et al*, 2008), as well as miR-10b (Malzkorn *et al*, 2010), have been reported to be downregulated in metastasis when compared with primary tumours. These miRNAs may have a role in ‘common’ progression/metastatic pathways that are used by a number of different cancers.

The miRNAs identified in our study have the potential to serve as prognostic markers in RCCs. Recently, it was shown that tumour-associated circulating miRNAs are elevated in the blood of breast cancer patients and they were associated with tumour progression (Roth *et al*, 2010). MicroRNAs have also been shown to be potential urinary markers for bladder cancer (Hanke *et al*, 2009). In addition, miRNAs were proved to be very stable and resistant to RNAse activity, extreme pH and temperature in body fluids (Chen *et al*, 2008; Hanke *et al*, 2009). The use of serum and urine markers is very attractive for RCC patients as the kidney is in direct contact with blood and urine.

In tumour progression and metastasis, miRNAs can act as oncogenes or tumour suppressors depending on their target genes. Gene regulation by miRNAs is not a simple process as they can have both direct and indirect targets. Direct targets of miRNAs can be affected at the mRNA level as a result of mRNA degradation or at the protein level by translation inhibition. Indirect targets of miRNAs can be downstream effector molecules of miRNA targets.

We found that increased expression of miR-215 decreased cell migration in RCC cells. This may be attributed to the effect of miR-215 on the expression of SIP1/ZEB2. SIP1/ZEB2 is a predicted direct target of miR-215 and we showed that upon transfection with miR-215, protein expression of this target was decreased. SIP1/ZEB2 represses E-cadherin and its role in epithelial-to-mesenchymal transition and more recently, the involvement of miRNAs in this interaction has been documented in the literature (Korpal and Kang, 2008). In a recent study, miR-192/215 was shown to repress SIP1/ZEB2 translation (Wang *et al*, 2010). Furthermore, Braun *et al* (2008) found that miR-192 and miR-215 had an effect on cellular adhesion and induced cell detachment. In addition, our metastatic gene profiler assay identified a number of genes that are involved in cell adhesion to be affected by miR-215, including cadherin 1 and integrin. These may be direct or indirect targets of miR-215. Through our metastatic gene profiling assay, we also identified a number of genes that are involved in the degradation of the extracellular matrix and are affected by increased miR-215 expression, including *MMP7* and *MMP13*. We also showed that miR-215 dysregulation directly affects cellular invasion in a cell line model. These data together support the role of miR-215 in tumour progression and metastasis. In addition to their clinical implications as prognostic markers, miRNA dysregulated in RCC metastasis reveals a new dimension to our understanding to the regulatory mechanisms of tumour progression and metastasis (Sotiropoulou *et al*, 2009; White and Yousef, 2010; White *et al*, 2011b). MicroRNAs also represent attractive therapeutic targets.

We showed that miR-215 expression was decreased in RCC metastatic samples when compared with primary RCC tissues in both fresh-frozen and FFPE tissues (Figure 2). miR-215 has also been shown to be downregulated in primary RCCs (White *et al*, 2011a) and colon cancers (Braun *et al*, 2008). The link between miR-215 and cell proliferation is currently being investigated and results suggest that miR-192 and miR-215 can induce cell-cycle arrest by increasing levels of p21 (Braun *et al*, 2008). Furthermore, miR-192 and miR-215 have been shown to be involved in chemosensitivity, and recent data suggest that expression levels may be a predictive marker for 5-fluoruracil treatment in colon cancer patients (Boni *et al*, 2010).

In conclusion, we identified a miRNA signature that characterises metastatic RCCs from primary RCCs. We also showed that miR-215 is a tumour suppressor in RCC metastasis. Through experimental analysis, we showed that overexpression of miR-215 in an RCC cell line model can affect metastatic potential through cellular migration and invasion. Through target prediction analysis and gene expression profiling, we also identified potential mechanisms and pathways in which miR-215 can have this effect. A better understanding of the mechanisms of metastasis will allow for the development of potential future therapeutic targets for RCC patients.

## Figures and Tables

**Table 1:** Significantly dysregulated miRNAs in metastatic *vs* primary clear cell renal cell carcinoma

**miRNA**	**Fold change metastatic/primary**	***P*-value**
*Upregulated*		
hsa-miR-638	2.380	0.0014239
hsa-miR-1915	2.271	0.0156326
hsa-miR-149^*^	2.149	0.009039
hsa-miR-1469	2.116	0.0035103
hsa-miR-200c	2.098	0.0138865
hsa-miR-720	1.947	0.0217099
hsa-miR-663	1.871	0.0004871
hsa-miR-498	1.449	0.0052433
hsa-miR-197	1.082	0.0132738
		
*Downregulated*		
hsa-miR-10b	0.183	2.90E-06
hsa-miR-196a	0.191	1.00E-07
hsa-miR-27b	0.273	1.37E-05
hsa-miR-29c	0.279	0.000174
hsa-miR-27a	0.287	7.62E-05
hsa-miR-204	0.288	0.0060097
hsa-miR-195	0.289	3.87E-05
hsa-miR-30a	0.296	3.90E-05
hsa-miR-192	0.301	0.0003271
hsa-miR-98	0.315	0.0035068
hsa-miR-126	0.334	4.00E-07
hsa-miR-143	0.337	9.57E-05
hsa-miR-30e	0.345	0.0003737
hsa-miR-15a	0.367	0.0004011
hsa-miR-26b	0.386	5.70E-06
hsa-miR-215	0.423	0.0072108
hsa-miR-30a^*^	0.426	0.0008579
hsa-miR-194	0.431	0.0009558
hsa-miR-10a	0.438	0.0100544
hsa-miR-200b	0.440	0.0008925
hsa-miR-30d	0.449	1.93E-05
hsa-miR-145	0.455	0.0009991
hsa-miR-29a	0.459	0.0015364
hsa-miR-30c	0.466	0.0268396
hsa-miR-455-3p	0.467	0.0005517
hsa-miR-181a	0.472	0.0003763
hsa-let-7e	0.489	0.0001903
hsa-let-7g	0.492	4.00E-07
hsa-miR-20a	0.500	0.0015265
hsa-miR-122	0.517	0.0425357
hsa-miR-24	0.525	0.0002439
hsa-miR-374b	0.534	0.0059959
hsa-miR-30b	0.543	0.029005
hsa-let-7f	0.559	2.45E-05
hsa-miR-151-5p	0.568	0.0009097
hsa-miR-361-5p	0.582	4.29E-05
hsa-miR-17	0.586	0.009408
hsa-miR-181b	0.590	0.0042813
hsa-miR-106a	0.592	0.0043882
hsa-let-7d	0.618	2.81E-05
hsa-miR-152	0.627	0.0048502
hsa-miR-26a	0.650	0.0008154
hsa-let-7a	0.659	0.0004721
hsa-miR-103	0.675	0.0256554
hsa-miR-30e^*^	0.683	0.0273815
hsa-let-7c	0.689	0.0004237
hsa-let-7i	0.701	0.0025987
hsa-miR-107	0.702	0.0296412
hsa-miR-106b	0.707	0.0337003
hsa-miR-29b	0.728	0.0399051
hsa-miR-23b	0.739	0.042895
hsa-miR-130a	0.752	0.0037552
hsa-miR-151-3p	0.760	0.0092847
hsa-miR-28-5p	0.771	0.0355917
hsa-let-7b	0.779	0.0097388
hsa-miR-200a	0.822	0.0540674

Abbreviation: miRNA=microRNA.

**Table 2:** The involvement of miRNAs dysregulated in metastatic renal cell carcinoma in the progression and metastasis of other cancers and their biological roles and targets

**microRNA**	**Dysregulation**	**Cancer**	**Biological function**	**Targets**
miR-204	Down	HNSCC	Adhesion, cell migration and invasion	SHP2
miR-196a	Up	Colorectal	Cell migration and invasion	HoxA7, HoxB8, HoxC8, HoxD8
miR-10b	Up	Breast	Cell migration and invasion	Hox D10, RhoC (through HoxD10)
	Up	Oesophageal	Cell motility and invasion	KLF4
	Up	Glioma	Cell invasion	uPAR, RhoC (through HoxD10)
miR-663	Down	Gastric	Prevents cell proliferation	Cyclin B
miR-122	Down	Liver	Decreased viability	
	Down	Liver	Metastatic progression	ADAM17
	Down	Liver	Migration and invasion	
miR-98	Up	Lung		FUS1
miR-29c	Down	Nasopharengyl	Invasion	Extracellular matrix proteins

Abbreviations: HNSCC=head and neck squamous cell carcinoma; miRNA=microRNA.

**Table 3:** Differential gene expression of metastasis-related genes upon miR-215 overexpression in kidney cancer cell lines

**Cell process**	**Unigene**	**Symbol**	**Description**	**Fold regulation**
Cell adhesion	Hs.461086	*CDH1*	Cadherin 1, type 1, E-cadherin (epithelial)	−1.549
	Hs.116471	*CDH11*	Cadherin 11, type 2, OB-cadherin (osteoblast)	1.522
	Hs.171054	*CDH6*	Cadherin 6, type 2, K-cadherin (fetal kidney)	2.633
	Hs.524484	*ITGA7*	Integrin, *α*7	−1.467
	Hs.449909	*RPSA*	Ribosomal protein SA	−1.503
	Hs.534797	*CTNNA1*	Catenin (cadherin-associated protein), *α*1, 102kDa	1.200
				
Extracellular matrix	Hs.2936	*MMP13*	Matrix metallopeptidase 13 (collagenase 3)	1.270
	Hs.375129	*MMP3*	Matrix metallopeptidase 3 (stromelysin 1, progelatinase)	−1.394
	Hs.2256	*MMP7*	Matrix metallopeptidase 7 (matrilysin, uterine)	1.461
	Hs.644633	*TIMP3*	TIMP metallopeptidase inhibitor 3	1.379
	Hs.44227	*HPSE*	Heparanase	−1.251
				
Cell-cycle regulation	Hs.202453	*MYC*	V-myc myelocytomatosis viral oncogene homolog (avian)	−1.200
				
Cell proliferation	Hs.160562	*IGF1*	Insulin-like growth factor (somatomedin C)	−1.484
	Hs.251526	*CCL7*	Chemokine (C-C motif) ligand 7	−1.598
	Hs.478275	*TNFSF10*	Tumour necrosis factor (ligand) superfamily, member 10	2.126
	Hs.523329	*EPHB2*	EPH receptor B2	−1.043
	Hs.279522	*NR4A3*	Nuclear receptor subfamily 4, group A, member 3	−1.257
	Hs.494178	*RORB*	RAR-related orphan receptor B	1.573
	Hs.514451	*SSTR2*	Somatostatin receptor 2	−1.377
	Hs.160411	*TSHR*	Thyroid-stimulating hormone receptor	1.370
	Hs.155942	*TRPM1*	Transient receptor potential cation channel, subfamily M, member 1	1.252
	Hs.208229	*KISSR1*	KISS1 receptor	−1.192
	Hs. 165950	*FGFR4*	Fibroblast growth factor receptor 4	−1.197
				
Transcription factor	Hs.12253	*SMAD2*	SMAD family member 2	−1.232
				
Other genes	Hs. 143212	*CST7*	Cystatin F (leukocystatin)	−1.195
related to	Hs.632466	*CTSK*	Cathepsin K	1.224
metastasis	Hs.95008	*KISS1*	KISS-1 metastasis-suppressor	1.206
	Hs.444986	*METAP2*	Methionyl aminopeptidase 2	−1.387

Abbreviation: miRNA=microRNA.

## Supplementary Material

Supplementary Figure 1A

Supplementary Figure 1B
