# Supplementary figures and images for: miRNA profiling in metastatic renal cell carcinoma reveals a tumour-suppressor effect for miR-215
Source: Br J Cancer. 2011 Oct 27;105(11):1741–9. doi: 10.1038/bjc.2011.401 (PMC3242591; doi:10.1038/bjc.2011.401)

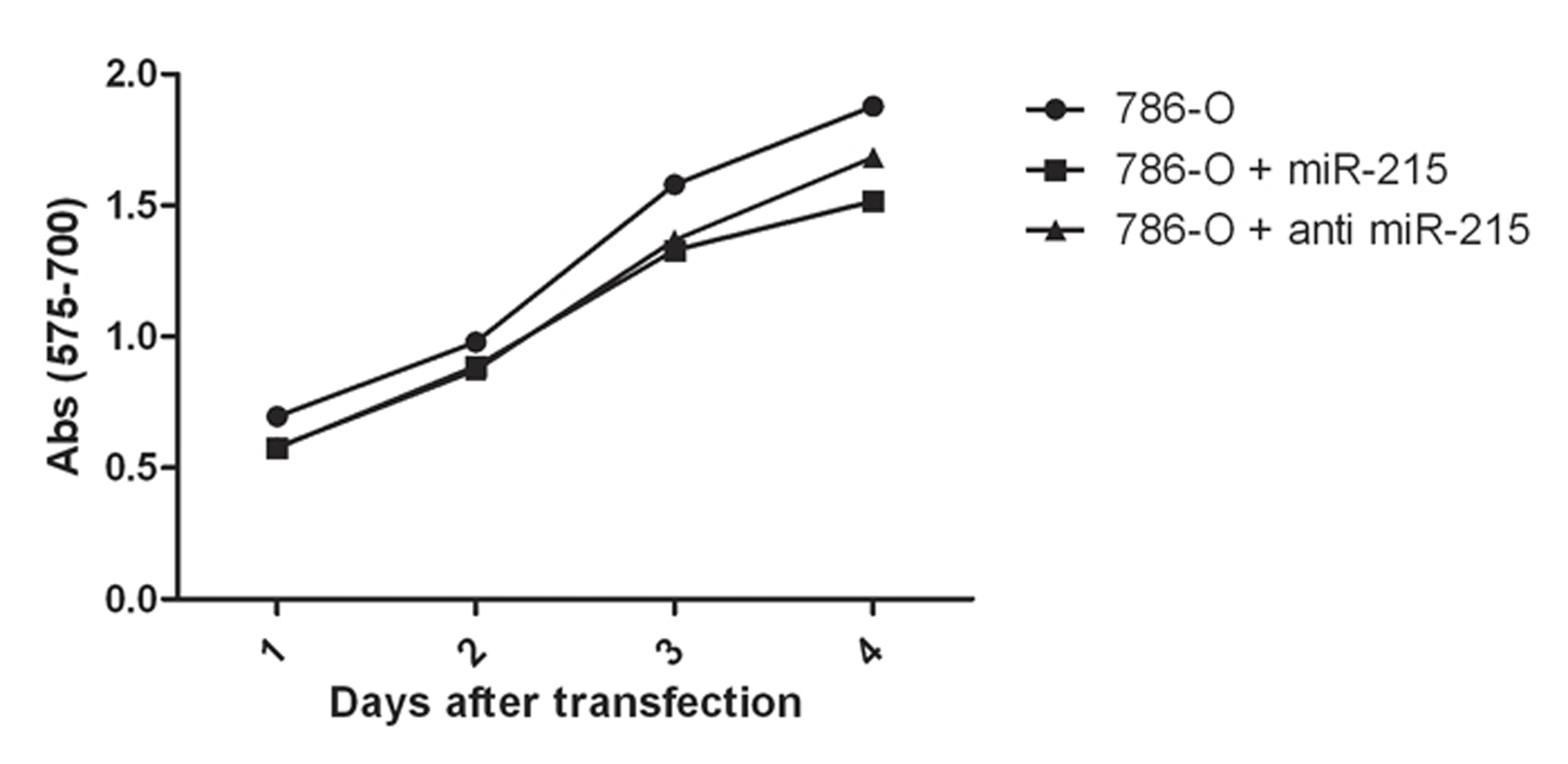

Supplement: Supplementary Figure 1A [file bjc2011401x1.tif]

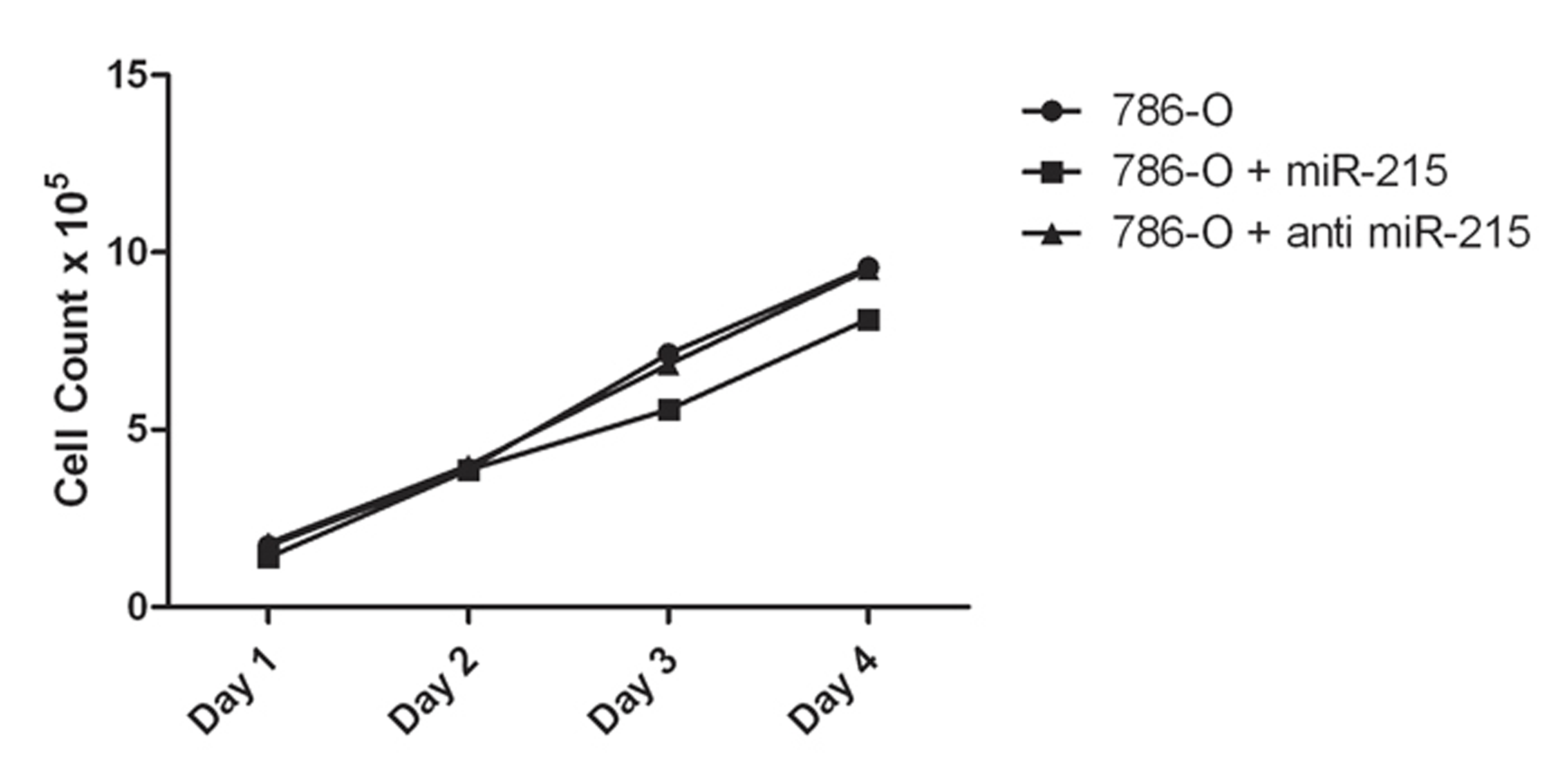

Supplement: Supplementary Figure 1B [file bjc2011401x2.tif]
