# Supplementary material for: miRNA profiling in metastatic renal cell carcinoma reveals a tumour-suppressor effect for miR-215
Source: Br J Cancer. 2011 Oct 27;105(11):1741–9. doi: 10.1038/bjc.2011.401 (PMC3242591; doi:10.1038/bjc.2011.401)
Supplement: Supplementary Figure Legend [file bjc2011401x3.doc]

**Supplementary Figure Legend**

**Supplementary Figure 1.** **miR-215 decreases cell proliferation in RCC cells.** (A) 786-O cells were transfected with miR-215 and cell growth was measured by MTT assay each day for five days after transfection. Cells that were transfected with miR-215 showed decreased metabolic activity when compared to untransfected cells. (B) 786-O cells were transfected with miR-215 and cell proliferation was measured by counting the number of cells after transfection. Cells transfected with miR-215 showed decreased cell proliferation when compared to untransfected cells.
