# Supplementary material for: miRNA profiling in metastatic renal cell carcinoma reveals a tumour-suppressor effect for miR-215
Source: Br J Cancer. 2011 Oct 27;105(11):1741–9. doi: 10.1038/bjc.2011.401 (PMC3242591; doi:10.1038/bjc.2011.401)
Supplement: Supplementary Table 1 [file bjc2011401x4.doc]

**Supplementary Table 1.** A partial list of the predicted targets of miRNAs that are dysregulated in metastatic RCC and their potential roles in tumor progression and metastasis.

| **Function** | **Target** | **Targeting miRNAs** |
| --- | --- | --- |
| **Cell Adhesion** | *CD44* | miR-15a, miR-27a, miR-27b |
|  | *CDH6* | miR-143 |
|  | *CDH11* | miR-15a, miR-26b, miR-27a,miR-27b, miR-130a, miR-181b,miR-186, miR-194, miR-195, miR-200c |
|  | *MTSS1* | miR-15a, miR-26b, miR-29c, miR-103, miR-107, miR-130a, miR-181b, miR-194, miR-195, miR-200a, miR-200b |
|  | *SIP1/ZEB2* | miR-29a, miR-29b, miR-29c, miR-215 |
| **Cell proliferation** | *IGF1* | miR-26b,miR-27a, miR-27b, miR-30a, miR-361-5p |
|  | *NR4A3* | miR-10a, miR-10b, miR-17, miR-15a, miR-20a, miR-103, miR-106a, miR-106b, miR-107, miR-181a, miR-181b, miR-182, miR-195, miR-215 |
|  | *RORB* | miR-130a, miR-181a, miR-181b |
|  | *VEGFA* | miR-29a, miR-29b, miR-29c, miR-126, miR-361-5p |
|  | *RB1* | let-7d, let-7e, let-7f, let-7g, miR-20a, miR-26b, miR-98,miR-106a, miR-106b, miR-143, miR-192, miR-215 |
| **Transcription Factor** | *MYCL1* | miR-29a, miR-29b, miR-29c, miR-93, miR-106b, miR-195, miR-638 |
|  | *SMAD2* | miR-30a, miR-455-3p |
|  | *SMAD4* | miR-26b, miR-28-5p, miR-204, miR-498 |
| **Matrix Metalloproteinase** | *MMP13* | miR-27a, miR-27b |
| **MMP inhibitor, Induction of apoptosis** | *TIMP3* | miR-15a,miR-20a, miR-20b,miR-30a, miR-30a*, miR-30b, miR-30c,miR-30d, miR-101,miR-103, miR-106a, miR-106b, miR-107, miR-143, miR-181a, miR-181b, miR-195, miR-200a |
